# Kinetic Studies on the Ability of Wines to Produce Hydrogen Sulfide (H_2_S) and Methanethiol (MeSH)

**DOI:** 10.1021/acs.jafc.5c04010

**Published:** 2025-07-28

**Authors:** Susana Ainsa-Zazurca, Ignacio Ontañón, Vicente Ferreira

**Affiliations:** Laboratorio de Análisis del Aroma y Enología, Química Analítica, Facultad de Ciencias, 16765 Instituto Agroalimentario de Aragón-IA2 (Universidad de Zaragoza-CITA), Zaragoza 50009, Spain

**Keywords:** reductive off-odors, sulfur compounds, polysulfides, persulfides, polythionates, glutathione

## Abstract

Twelve wines have been stored in anoxia to monitor hydrogen sulfide (H_2_S) and methanethiol (MeSH) emitted. Emissions were studied for 3 months at 75 °C, 10 months at 50 °C, and 18 months at 35 °C. H_2_S emissions followed first-order kinetics with half-lives of 0.89 months at 75 °C and (estimated) 24.6 and 160 months at 50 and 35 °C, respectively. Total H_2_S precursors ([*P*]_0_), calculated due to the exhaustion observed at 75 °C, amount to between 1.4 and 2.96 mg/L of H_2_S. [*P*]_0_ correlated with the brine-releasable H_2_S accumulated by the wines in accelerated reductive aging (AR). MeSH emissions hardly decreased and at 75 °C were between 0.36 and 0.82 mg/L, exceeding the decrease in free methionine (0.26 mg/L on average). MeSH emissions determine MeSH accumulation in AR, far less effectively in red wines, suggesting reactions with polyphenols. MeSH emissions negatively correlated with [*P*]_0_, suggesting that these play a key role in regulating redox chemistry during wine aging.

## Graphical abstract

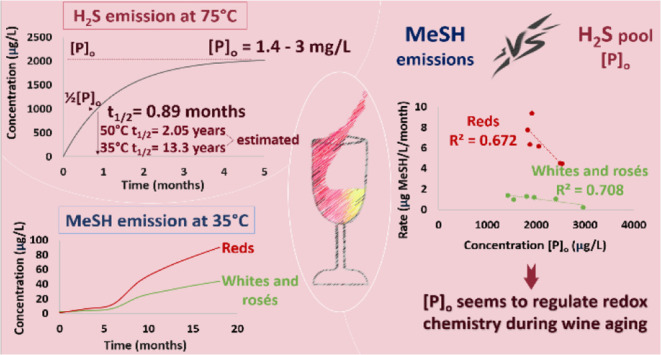

## 1 Introduction

The so-called “reductive” defect is an off-odor caused by the accumulation of H_2_S, MeSH, and, eventually, other mercaptans during the stage of wine in airtight vessels.^1–3^ It is a common problem,^4^ whose occurrence is increasing as winemakers tend to use techniques minimizing oxygen exposure during winemaking and storage.^5^ All of the wines naturally accumulate small levels of both components. In commercial wines, levels of H_2_S accumulated in one year were between 1.1 and 12 μg/L and those of MeSH were between 0.7 and 3.5 μg/L^3^. Problematic wines, which are obviously not commercialized, can accumulate much higher levels.^1^ The chemical causes behind these accumulations are not completely understood, although the present experimental evidence makes it possible to state that the major cause is the spontaneous reduction of organic polysulfides and hydropolysulfides.

The existence of organic disulfides and polysulfides able to release H_2_S and MeSH has been well documented in recent years.^6–8^ These structures can be formed from elemental sulfur present in the must,^9^ both by the action of metals and directly by yeasts. These produce polysulfides and hydropolysulfides as signaling agents,^10^ with marked differences between strains,^11^ and they can use both methionine (Met) and cysteine (Cys) as sulfur sources.^12^ So far, the existence of mixed polysulfides and hydropolysulfides of cysteine and glutathione (GSH) has been demonstrated^13^ even with the varietal polyfunctional mercaptans.^14^ The sulfur chains described have up to six S atoms, the most stable and abundant being those with three.^7^ Cys-S-sulfonates have been also found.^7^ The existence of polythionates and monosulfonic polysulfanes has been hypothesized,^15^ but not yet experimentally confirmed. However, a tetrathionate has been detected, but it seems unlikely to be a precursor of H_2_S.

In addition to this complex family of polysulfanes, MeSH can also originate from dimethyl disulfide (DMDS) and methylthioacetate^16^ and both H_2_S and MeSH can originate from metal-catalyzed desulfhydration of Cys and Met.^17^ Another possible reservoir of H_2_S and to a lesser extent MeSH are complexes with copper and other metals.^18,19^ These complexes act as a barrier to contain the proportion of free forms so that as long as metal cations are available, H_2_S and to a lesser extent MeSH formed become integrated into the complex fraction.^20^ When the metals are completely complexed, free forms of H_2_S and MeSH begin to accumulate, which is manifested by a drop in the redox potential.^21^

One of the problems in the study of reduction processes is the difficulty of knowing the real dimensions of the precursor pool, which appears to be much higher than previous estimates, at least when it is evaluated during accelerated aging according to recently presented evidence.^22^ In fact, the potential H_2_S levels measured by addition of a reductant and a metal-complexing agent are below 0.08 mg/L^6,23^ when it has been described that some wines could emit at 50 °C quantities above 0.35 mg/L in 2.5 months and quantities above 2 mg/L at 75 °C in one month.^22^

These results, if confirmed, would suggest that the fraction identified and quantified by HPLC-MS to date represents a small fraction of the existing ones and that further research should be carried out to elucidate the chemical nature of the precursors.

Because of this, the main objective of the present work is to measure the H_2_S and MeSH emission capacity of commercial wines when stored in anoxia. The study was carried out at three different temperatures (75, 50, and 35 °C) and for relatively long periods (3, 10, and 18 months, respectively) in order to be able to draw general conclusions.

## 2 Material and Methods

### 2.1 Reagents and Chemicals

Cysteine (99%), methionine (99%), glutathione (>98%), and α-aminobutyric acid (AABA, 98%) were from Sigma-Aldrich (Madrid, España). Purified water was obtained from a Milli-Q system (Merck, Molsheim, France). Phosphoric acid, hydrogen peroxide, methyl red, methyl blue, sodium hydroxide, sodium chloride, and ascorbic acid were from Panreac AppliChem (Barcelona, España). Acetonitrile (ACN) LC-MS grade was from Fisher (Seelze, Alemania). Copper­(I) chloride, tris­(2-carboxyethyl)­phosphine hydrochloride (TCEP), and ethylmethylsulfide (EMS) were from Sigma-Aldrich (MO, USA). Ethanol and ammonium formate (99%) were from Merck (Darmstadt, Alemania), and hydrochloric acid (HCl) was from Scharlau (Barcelona, España). The brine contained 350 g/L NaCl and 0.5 g/L ascorbic acid in Milli-Q water.

### 2.2 Samples and Chemical Characterization

Twelve different commercial wines were used. The basic information on sample codes, wine types, grape varieties, vintages, and geographical origins (designations of origin) is detailed in Table 1.

**Table 1:** Wines Analyzed in the Experiment, Including Varietal Composition, Age, and Origin and Some Basic Compositional Data

Sample code	Wine type	Grape variety	Vintage year	Denomination of origin	Alcohol (%. v/v)	pH	Free SO_2_ (mg/L)	Total SO_2_ (mg/L)	Redox Potential (mV)	Free H_2_S (μg/L)	Free MeSH (μg/L)
VT1	Red	Tempranillo	2021	Rioja	14.0	3.48	15.2 ± 1.1	44.8 ± 0	–27	0.110 ± 0.004	<LOD^a^
VT2	Red	Tempranillo	2021	Rioja	14.5	3.88	8.48 ± 1.58	18.9 ± 0.5	–47	2.56 ± 0.8	0.97 ± 1.32
VT3	Red	Tempranillo	2020	Ribera del Duero	14.5	3.60	10.4 ± 0.2	33.6 ± 3.2	–34	0.290 ± 0.002	<LOD^a^
VT4	Red	Garnacha and Syrah	2021	Cariñena	14.5	3.64	17.6 ± 2.3	65.6 ± 0	–29	0.83 ± 0.12	0.577 ± 0.77
VT5	Red	Garnacha	2019	Somontano	15.0	3.50	8.16 ± 0.23	54.6 ± 2.5	–27	1.73 ± 0.5	1.54 ± 0.11
VT6	Red	Syrah	2019	Somontano	14.5	3.55	<LOD^a^	8 ± 0	–43	6.83 ± 0.13	2.61 ± 0
VB7	White	Verdejo	2021	Rueda	13.0	3.25	11.4 ± 0.2	67.8 ± 6.3	–10	3.45 ± 0.05	0.94 ± 1.28
VB8	White	Macabeo and Chardonnay	2021	Somontano	13.0	3.28	15.2 ± 5.7	103 ± 3	–40	7.61 ± 5.85	2.28 ± 0.33
VB9	White	Gewürztraminer	2021	Somontano	12.5	3.46	8.16 ± 2.49	49.0 ± 0.9	–43	0.54 ± 0.39	5.44 ± 0.48
VR10	Rosé	Garnacha	2021	Campo de Borja	13.5	3.15	<LOD^a^	73.9 ± 1.8	–7.5	0.79 ± 0.42	1.36 ± 0.08
VR11	Rosé	Syrah and Cabernet	2021	Somontano	13.5	3.26	4 ± 6	35.5 ± 8.6	–83	8.44 ± 4.96	3.30 ± 1.16
VB12	White	Chardonnay	2021	Campo de Borja	13.0	3.32	4.80 ± 1.81	103 ± 5	–0.5	0.0152 ± 0.0152	<LOD^a^

a LOD-free SO_2_: 3.2 mg/L; LOD-free MeSH: 0.035 μg/L.

The wines were initially characterized by determining free and total SO_2_, pH, metals, cysteine, methionine, and glutathione as well as the free and BR forms of VSCs and redox potential, using a platinum electrode with Ag/AgCl as the reference. Additionally, accelerated reductive aging (AR) tests were performed.

Free and total SO_2_ were determined using the official Rankine method following OIV recommendations.^24^ The metal content (Zn, Cu, Fe, and Mn) was determined in triplicate using inductively coupled plasma mass spectrometry (ICP-MS) following the methodology described by Gonzálvez et al.^25^ The amino acids Cys, Met, and the peptide GSH were measured both in the initial samples and after 3 months of incubation at 75 °C. The analysis was performed using HPLC-MS. Briefly, inside the anoxia chamber (Jacomex, France), 880 μL of ACN and 20 μL of an internal standard (0.5 mM AABA) were added to 100 μL of the sample in a 2 mL Eppendorf tube. Then, 10 μL of the sample, filtered through a 0.22 μm Nylon filter, was injected into a Waters XBridge Amide column (150 mm × 2.1 mm I.D. × 3.5 μm) and separated using an aqueous gradient of ammonium formate at pH 3.0 (A) and ACN:water (90:10) with 10 mM ammonium formate (B) at a flow rate of 0.5 mL/min. The gradient was as follows: initial 100% B, 75% B at minute 7, 50% B at minute 8, and back to 100% B at minute 9.1 (hold 4 min). The triple quadrupole mass detector (QqQ) was an EVOQ LC-TQ, and the transitions used for the determination are shown in Table S1. Calibration was performed by adding known amounts of the three analytes to each sample and determining the corresponding sample-specific response factors.

The analysis of free and BR forms of H_2_S and MeSH was performed using the method proposed by Ontañon et al.,^19^ operating inside the anoxic chamber. Briefly, for the analysis of free forms, 12 mL of wine were placed in a 20 mL vial, 40 μL of IS (10 mg/L EMS in ethanol) were added, and the vial was sealed and then removed from the chamber for HS-GC-SCD analysis. One mL of headspace, thermostated at 30 °C, was injected in split 1:2 using a 1 mm inner diameter ultrainert liner and the cryofocusing system (Gerstel CTS 2) activated at −150 °C for 0.8 min. For BR forms, 1.8 mL of wine were mixed with 10.8 mL of brine and 40 μL of IS (2 mg/L EMS in ethanol). Then, 1 mL of headspace, thermostated at 70 °C, was injected in split 1:15 with the syringe at 80 °C. A 4 mm inner diameter ultrainert liner was used, and the cryofocusing system was deactivated. The instrument used was an Agilent 7890B with a Sulfur Chemiluminescence Detector 8355 (GC-SCD) and a Supelco SPB-1 SULFUR column (30 m × 0.32 mm I.D. × 4 μm film thickness), preceded by a 60 cm × 0.32 mm I.D. fused silica precolumn with polar deactivation. The injection was performed with a Combi-PAL autosampler from CTC Analytics (Zwingen, Switzerland) in a multimode injector (MMI) at 150 °C. The carrier gas was He. The detector temperature was 280 °C, and the burner temperature was 800 °C. The oxidizing gas flow rate was 50 mL/min, and the hydrogen flow rate was 38 mL/min in the “upper flow” and 7 mL/min in the “lower flow.”

The wines underwent AR following the method proposed by Franco-Luesma and Ferreira,^26^ with an incubation time of 2 weeks. After the accelerated aging process, free and BR forms were determined by using HS-GC-SCD under the chromatographic conditions previously described. The redox potential of the wine was evaluated in the initial samples and after AR. Measurements were performed inside the anoxic chamber using a HI198191 system (Hanna Instruments, RI, USA), with a signal stabilization time of 35 min as indicated elsewhere.^21^ Each sample was measured in duplicate.

### 2.3 Anoxic Storage and Measurement of H_2_S and MeSH Emissions

The device used to study the emission of sulfide gases was described and validated by Ferreira et al. (2023).^22^ It consists of a 100 mL flat-bottom round glass flask containing a 20 mL vial inside, which holds 10 mL of trapping solution. A total of 80 mL of the wine sample was placed in the main flask. The trapping solution was prepared by a 1:100 dilution of a stock solution containing 10 g/L CuCl in water with 1.5 M HCl. Three samples of each wine were prepared for each temperature condition and were stored for 18 months at 35 °C, 10 months at 50 °C, and 3 months at 75 °C. In the samples incubated at 35 °C, the trapping solutions were replaced and analyzed every 3 months; at 50 °C, monthly; and at 75 °C, every 1 to 2 weeks. For the analysis of gases absorbed in the trap, 0.5 mL of the trapping solution was added to a 20 mL vial containing 11.5 mL of brine. Thirty mg of TCEP and IS were then added, and the samples were analyzed following the procedure described for the analysis of BR forms of H_2_S and MeSH.

### 2.4 First-Order Kinetic Model to Interpret Experimental H_2_S

The emission functions at 75 °C were fitted to the exponential decay function of the H_2_S precursor pool, assuming first-order kinetics as follows:

$${[P]}_{t}={[P]}_{o}\times e^{-kt}$$

where [*P*]_*t*_ referred to the concentration of the pool of H_2_S precursors at time *t*, [*P*]_0_ referred to the concentration of such a pool at the beginning of the experiment, and *k* was the reaction rate constant. In order to transform into a linear function, data were transformed into natural logarithms so that

$$\ln{[P]}_{t}=\ln{[P]}_{o}-kt$$

With the following considerations and assumptions:

The concentration of the pool of H_2_S precursors contained in a given wine was expressed as the concentration of H_2_S that such a wine can emit. Then, it followed that [*P*]_0_ = [H_2_S]_total_, where [H_2_S]_total_ was the concentration of H_2_S that the wine was able to emit from the beginning of the experiment.
[*P*]_*t*_, the concentration of precursors remaining at time *t,* was the concentration of H_2_S that such a wine was able to emit from time *t* to infinity. This made it possible to establish that [*P*]_*t*_ = [*P*]_0_ – [H_2_S]_*t*_, where [H_2_S]_t_ was the concentration of H_2_S emitted from the beginning of the experiment to time *t*. These are the concentrations represented in Figure 1e,f. Also, [*P*]_*t*_ = [H_2_S]_total_ – [H_2_S]*_t_.*
[*P*]_0_ and [H_2_S]_total_ were obtained from experimental data as the total concentrations of H_2_S at which the plots shown in Figure 1e,f converged. As a first approximation, we found that [H_2_S]_total_ was 1.08 times the amount accumulated in the three months of the study.
Then, ln­[*P*]_*t*_, obtained as ln­([H_2_S]_total_ – [H_2_S]_*t*_), was represented versus time, and experimental data were fitted to the least-squares linear regression model, whose slopes were −*k,* and the intercepts were (ln­[*P*]_0_). Then, the validity of the assumptions and the model were checked, and, if required, the value initially assigned to [H_2_S]_total_ was modified to improve the fitting.
Finally, it was assumed that all H_2_S precursors form H_2_S through a first-order reaction with the same rate constant.

**Figure 1:**
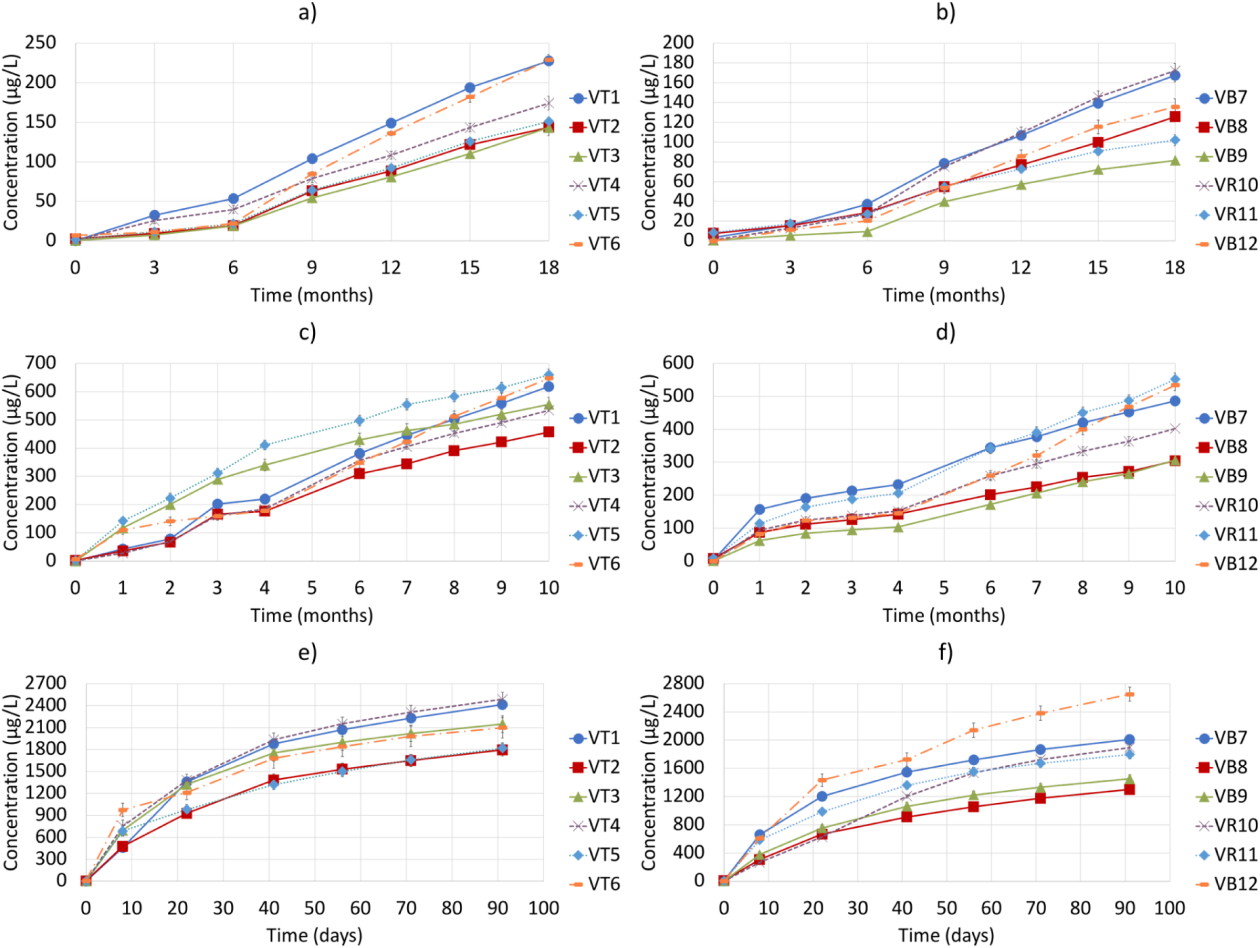
Accumulated H_2_S emitted by the 12 wines at 35 °C (a and b), 50 °C (c and d), and 75 °C (e and f). Plots on the left (a, c, and e) correspond to red wines, while those on the right (b, d, and f) correspond to whites and rosés. Error bars are mean errors.

## 3 Results and Discussion

Some basic characteristics of the 12 commercial wines used in the study can be seen in Table 1, while other chemical information can be seen in Tables S2, S3, and S4. Selected samples were normal commercial wines, all of which had spent several months in the bottle at the beginning of the study, which explained that initial redox potentials were in most cases negative and that some of them had already accumulated detectable levels of H_2_S and MeSH.

### 3.1 Emission of H_2_S During Wine Anoxic Storage

The plots representing the accumulated emissions of H_2_S by the 12 wines during their anoxic storage at three different temperatures can be seen in Figure 1, while the different mathematical models used for fitting the experimental data are summarized in Table 2.

**Table 2:** Summary of Models Used to Interpret the Emission of H_2_S During the Anoxic Storage of Wines at Three Different Temperatures

	35 °C (6 months to 18 months)		50 °C (1 month to 4 months)		50 °C (4 months to 10 months)		75 °C			
H_2_S	*R* ^2^	Slope	*R* ^2^	Slope	*R* ^2^	Slope	*R* ^2^	*k* (−slope) (months^–1^)	*t*_1/2_ (months)	*P*_o_ (e^∧^(intercept)) (μg/L)
VT1	0.995	14.6	0.917	65.4	0.992	65.2	0.994	0.838	0.827	2527
VT2	0.987	10.2	0.911	52.3	0.974	45.7	0.996	0.828	0.837	1866
VT3	0.998	10.2	0.988	75.7	0.989	34.7	0.994	0.949	0.730	2048
VT4	0.998	11.1	0.950	56.0	0.963	56.5	0.997	0.827	0.838	2488
VT5	0.992	10.7	0.998	89.6	0.992	40.9	0.990	0.692	1.002	1820
VT6	0.996	17.0	0.980	22.3	0.998	78.6	0.989	0.908	0.763	1911
VB7	0.995	10.7	0.983	25.2	0.986	41.6	0.994	0.812	0.854	1957
VB8	0.999	7.98	0.985	18.0	0.996	26.4	0.989	0.811	0.854	1415
VB9	0.955	5.88	0.947	13.3	0.998	33.3	0.997	0.825	0.840	1540
VR10	0.990	12.0	0.954	18.7	0.987	40.8	0.996	0.591	1.172	2399
VR11	0.977	6.19	0.942	29.9	0.993	56.3	0.997	0.824	0.841	1801
VB12	0.993	9.75	0.886	19.8	0.997	65.8	0.991	0.598	1.160	2960
	Global Average	10.5		40.5		48.8		0.792	0.893	2061
	Reds Av	12.3		60.2		-		-	-	-
	Wt&Rs Av	8.75		20.8		-		-	-	-

Emissions at 35 °C are presented in Figure 1a (red wines) and Figure 1b (whites and rosés). Average levels accumulated by the wines over the 18 months of observation were 154 ± 12.7 μg/L (8.6 μg/L per month). The difference between the highest-emitting wine, VT6, which emitted 229 μg/L, and the lowest-emitting wine, VB9, which emitted 81.4 μg/L, was a factor of 2.8. Differences between red and white were not significant. In the first 6 months, the average emission was 4.5 μg/L/month, with the minimum corresponding to VB9 with 1.6 mg/L/month and the maximum to VT1 with 8.9 μg/L/month (×5.6 difference). From that time on, emissions seemed to stabilize and followed an approximately linear behavior so that accumulated emissions between months 6 and 18 were adjusted by least-squares regression (see Table 2). As shown in Table 2, the average production in this period was 10.5 μg/L/month—12.3 for reds and 8.75 for whites and rosés (difference significant at *p* < 0.05). The maximum emission was that of red VT6 with 17.0 μg/L/month, and the minimum was that of white VB9 with 5.88 μg/L/month (×2.9 difference).

Emissions at 50 °C are shown in the plots displayed in Figure 1c,d. In this case, the maximum emission was observed in the first month, in which, on average, 89.2 μg/L of H_2_S were emitted. Thereafter, emissions stabilized, and two different relatively linear trends were observed. The first one was between months 1 and 4, and the second between months 4 and 10. Between months 1 and 4, emissions in white wines slowed (20.8 μg/L/month on average) and were much lower than those in red wines (60.2 μg/L/month on average). Between months 4 and 10, the differences between whites and reds disappeared, and an average emission of 48.8 μg/L/month was reached, with a minimum of 26.3 in VB8 and a maximum of 78.6 in VT6 (×3 difference). At the end of the period, the amount of H_2_S emitted by the average wine was 504.8 μg/L—red wines emitted 578.6 μg/L, compared to 431.0 μg/L for white wines (difference significant at *p* < 0.05). The maximum emitted was 659.9 μg/L in VT5, and the minimum was 304.6 μg/L in VB8 (×2.2 difference).

Finally, Figure 1e,f shows H_2_S emission at 75 °C. In this case, the rate of change of the curves decreases to approach zero, suggesting depletion of the H_2_S precursor pool. The average amount emitted in the first 8 days (570 μg/L) corresponded to an emission per month of 2.1 mg/L, while that emitted in the last 21 days corresponded to 0.66 mg/L/month. The average total emitted in the period studied was 1.99 mg/L, with no significant differences between reds, whites, and rosés. The wine that accumulated the most was VB12 with 2.65 mg/L, and the least was VB8 with 1.30, only twice less.

The assumptions, detailed in Section 2.4, allowed us to obtain the functions modeling the accumulated amounts of H_2_S emitted by each one of the wines (i.e., the experimental plots given in Figure 1e,f) as

$${[H_{2}S]}_{t}={[H_{2}S]}_{total}\left(1-e^{-kt}\right)$$

The results of the model can be compared in Table 2 and Figure S1. As can be seen, the fittings were in all cases very good, with *R* ^2^ better than 0.989 and in 8 cases better than 0.994. The reaction rate constants, expressed in months^–1^, ranged from 0.591 to 0.949, with 0.792 as the average. Average half-lives (ln(2)/*k*) ranged from 0.73 to 1.17 months, with 0.893 as the average half-life. No clear relationship was observed between the reaction rate constants and pH. A significant negative correlation with the total SO_2_ levels (significant at *p* < 0.05) was observed. There were also no differences in the constants between reds and whites.

The [*P*]_0_ values estimated by the models represented the total H_2_S precursor concentrations, which allowed us to establish that the wines contained between 1.41 mg/L (VB8) and 2.96 mg/L (VB12) H_2_S precursors (expressed as H_2_S), with a mean of 2.06 mg/L and a deviation of 0.45 mg/L, with no differences between reds and whites and rosés. The agreement between the *P*_o_ values estimated through the model and the initial assumptions was quite good. Both sets of values were correlated with a slope not significantly different from 1 and an RMS of 109, less than 5.0%.

### 3.2 H_2_S Emission Rate and Temperature

This study was carried out with the average accumulated emitted amounts of H_2_S by the 12 wines, taking as a reference the average accumulated emission curve at 75 °C represented by the following equation:

$${[H_{2}S]}_{t}=2061\times \left(1-e^{-0.792t}\right)$$

It could be seen that the average accumulated emissions after 10 months at 50 °C were reached in 0.355 months at 75 °C, and those observed after 18 months at 35 °C would be reached in 0.00432 months. This suggested that emission at 50 °C was 28.17 times slower than that at 75 °C and at 35 °C, 183 times slower, and showed that kinetics were extremely temperature-dependent. This simple calculation suggested that the average *k* at 50 °C was 0.0281 month^–1^ and at 35 °C was 0.00433 month^–1^. Plotting the logarithms of these constants against the inverse of the temperature in degrees Kelvin yielded a linear representation of the equation log­(*k*) = 17.39–6096.7 × 1/*T* with a reasonable fit (*R* ^2^ = 0.9974, significant at *p* < 0.05). This made it possible to estimate that *k* at 25 and 20 °C would be 0.000855 and 0.000383, respectively. The estimated half-lives would be 2.05 years at 50 °C, 13.3 years at 35 °C, 67.6 years at 25 °C, and 150 years at 20 °C. These estimates, although crude, explained that neither at 35 °C nor at 50 °C were there any signs of depletion of the precursor pool and showed that at room temperature the wine is a continuous emitter of small amounts of H_2_S, even well past a typical storage time for a wine. Apparent activation energy was 50.7 kJ/mol, which is a similar value to the activation energies obtained in other wine chemistry reactions, for example, ester hydrolysis, with 29 and 59 kJ/mol for 2-phenylethyl acetate and isoamyl (plus active amyl) acetate, respectively, in a Pinot noir and 64 kJ/mol for isoamyl (plus active amyl) acetate in a Chardonnay.^27^

It could be estimated that the average amounts of H_2_S emitted in one year at 25 °C were 21 μg/L, consistent with the 6.2 μg/L average increase of the accumulated free amounts after one year of aging at 25 °C,^3^ assuming that a fraction of what is emitted reacts with other wine components and does not accumulate. The fact that the accumulated amounts found in that report^3^ were significantly lower in red wines, when emissions from red wines were higher for shorter time points (1–4 months at 50 °C) (Table 2), should be attributed precisely to the higher presence of polyphenols with electrophilic characteristics in these wines, confirming recent observations.^28^

### 3.3 H_2_S Emissions and Accumulation and Dimensions of the Pool, [*P*]_0_

H_2_S emissions at 35 °C from the sixth month onward were positively and significantly correlated with the [*P*]_0_ × *k* product (*R* ^2^ = 0.336–0.351, *p* < 0.05). On the other hand, H_2_S emissions in the first 3 and 6 months were positively and significantly correlated with the GSH content of the wine (*R* ^2^ = 0.379 and 0.402, *p* < 0.05). Empirically, it was also verified that H_2_S emissions at 35 °C in all the time periods studied were positively and significantly correlated with the product [*P*]_0_ × *k* × SQR (GSH) (*R* ^2^ = 0.566–0.360, *p* < 0.05). These results suggested that the size and stability of the precursor pool are primary determinants of the rate at which H_2_S is emitted at 35 °C from the sixth month onward. The facts that the explained variances were not higher and that emissions were explained only from the sixth month onward are probably a consequence of the fact that the precursor pool is still an amalgam of diverse structures whose individual reduction kinetics will differ, as will their distribution between wines. The correlation for the released H_2_S at the final time point for 35 and 75 °C (*R* ^2^ = 0.293, *p* = 0.0694) also supported this hypothesis. In this sense, it should be considered that the estimates obtained at 75 °C for [*P*]_0_ represent a cumulative value of H_2_S formed from all precursors under elevated temperatures, and *k* is a weighted average value (weighted by the relative concentration of each precursor), so it is to be expected that the emissions at 35 °C of the different wines will be more or less distant from the [*P*]_0_ x *k* product depending on their particular distribution of precursors. In fact, the correlation with GSH could be due to the fact that wines with more free GSH could have a higher proportion of GSH-ending polysulfides, and these could be the first to hydrolyze. In any case, the existence of these relationships would underpin the validity of both [*P*]_0_ and *k* values obtained in the present work.

On the other hand, the development of the reduction defect in wines is a consequence of the accumulation of H_2_S during anoxic aging, and this accumulation must be the result of both the wine’s capacity to emit H_2_S and to avoid its accumulation by reactions with electrophilic components.^29^ The tendency of the wines to develop reductive off-odors is evaluated by means of an accelerated reduction test,^26^ in which the free and BR H_2_S accumulated after anoxic storage at 50 °C are measured. The amount of H_2_S BR accumulated in this test by the wines was positively and significantly correlated with [*P*]_0_ (*R* ^2^ = 0.614, *p* < 0.01) (Figure S2). The accumulated free amount was on its side significantly correlated with the amounts emitted at 3, 6, 9, 12, 15, or 18 months at 35 °C (all *R* ^2^ > 0.342, *p* < 0.05, with the best at 9 months, *R* ^2^ = 0.568, *p* < 0.01) (Figure S3), failing the correlation with [*P*]_0_ due to the specific behavior of VB12 wine. These results showed that [*P*]_0_ plays an essential role in the development of the reduction defect, in which the emission process is a determinant.

### 3.4 Emission of Mesh During Wine Anoxic Storage

The MeSH emission curves at the three temperatures studied can be seen in Figure 2, and the different data from the models used to adjust these curves can be seen in Table 3 and Figure S4.

**Figure 2:**
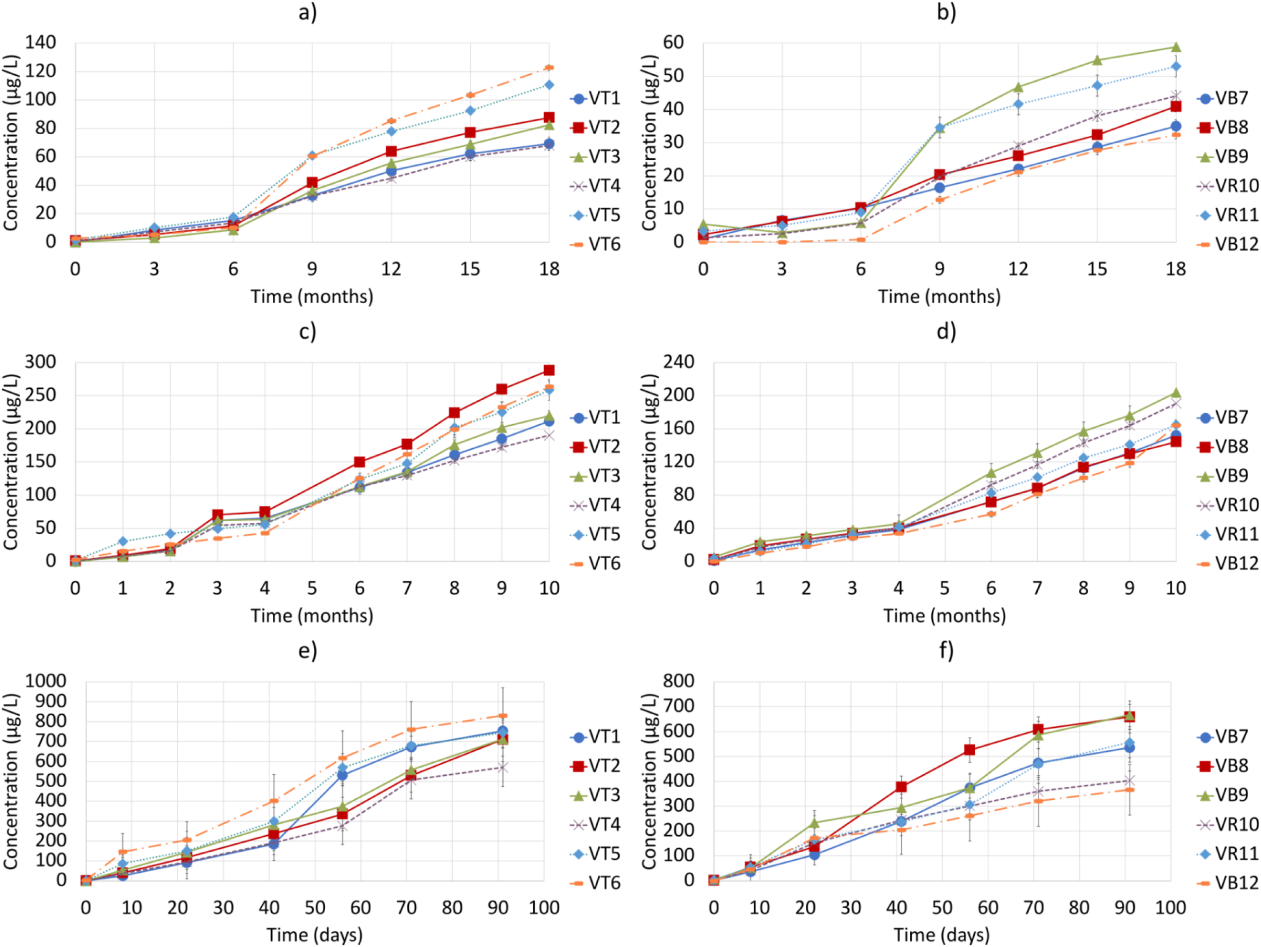
Accumulated MeSH emitted by the 12 wines at 35 °C (a and b), 50 °C (c and d), and 75 °C (e and f). Plots on the left (a, c, and e) correspond to red wines, and those on the right (b, d, and f) correspond to whites and rosés. Error bars are mean errors.

**Table 3:** Summary of Models Used to Interpret the Emission of Mesh During the Anoxic Storage of Wines at Three Different Temperatures

	35 °C (9 months to 18 months)		50 °C (4 months to 10 months)	
MeSH	*R* ^2^	Slope	*R* ^2^	Slope
VT1	0.965	4.04	0.999	24.4
VT2	0.970	5.02	0.997	36.1
VT3	0.991	5.08	0.991	27.2
VT4	0.986	4.04	0.992	21.8
VT5	0.998	5.47	0.993	34.3
VT6	0.995	6.84	0.997	36.8
VB7	0.999	2.08	0.996	19.1
VB8	0.991	2.27	0.994	17.9
VB9	0.950	2.71	0.994	25.9
VR10	0.991	2.75	0.999	25.1
VR11	0.997	2.03	0.999	20.4
VB12	0.984	2.18	0.949	20.8
	Global Av	3.71		25.8
	Reds Av	5.08		30.1
	Wt&Rs Av	2.34		21.5

At 35 °C, and as observed in the case of H_2_S, emissions during the first 6 months were very low. Emissions were activated only after the sixth or ninth month, depending on the wine. In any case, it can be seen that the emissions of reds were clearly higher than those of whites. In the first 6 months, an average of 9.9 μg/L (1.65 μg/L/month) was emitted, with a maximum of 17.6 (VT5) and a minimum of 0.75 (VB12). Red wines emitted on average 12.8 compared to 7.0 for white wines (*p* < 0.05). The period from 9 to 18 months was approximated by a straight line, which showed (Table 3) that red wines emitted 5.08 μg/L/month on average, against only 2.3 for white wines (*p* < 0.001). The maximum monthly emission was 6.84 μg/L/month (VT6), and the minimum was 2.03 μg/L/month (VR11).

At 50 °C, the emission curves had a first part of low emission, more irregular for the reds, up to month 4, and from then on, a constant emission was observed in all cases. In the fourth month, the reds emitted an average of 60 μg/L (15 μg/L/month), compared to only 39.7 μg/L (10 μg/L/month) for the whites (significant difference, *p* < 0.01). Between months 4 and 10, all emitted fixed levels that could be fitted to a straight line with determination coefficients better than 0.991 in all cases (see Table 3) except for VB12, which had a higher emission in the last month (Figure 2f). In this period, red wines emitted 30.1 μg/L/month on average, significantly more than whites, which emitted 21.5 μg/L/month (*p* < 0.05). In any case, the differences between the smallest emitter (VB8 with 17.9 μg/L/month) and the strongest one (VT6 with 36.8 μg/L/month) hardly exceed a factor of 2.

Finally, MeSH emissions at 75 °C are shown in Figure 2e,f. In this case, we found a more erratic behavior due to the larger imprecision of the method^22^ and a greater diversity of behaviors, which made modeling difficult. In all cases, emission peaks were observed, more or less pronounced, depending on the wine, at one or even two sampling points. In any case, except in VT2 and VT3 wines, the emission rates at the last sampling point were the lowest or very close to the lowest, suggesting that after reaching clear maxima, MeSH emission began to slow down, although it was far from ceasing. The mathematical fitting to decay functions was not possible. The total amounts of MeSH emitted over the three months were significantly higher in reds (720 μg/L) than in whites (531 μg/L) (*p* < 0.05). The lowest emission was 366 μg/L (VB12), and the highest was 830 μg/L (VT6).

Emissions at the three temperatures were well correlated. Specifically, emissions at 35 °C from month 6 were well correlated with total emissions at 75 °C (*R* ^2^ = 0.435, 0.625, 0.625, 0.662, 0.644, 0.643). They were also well correlated with accumulative emissions at 50 °C from month 6 onward.

### 3.5 MeSH Emission Rate and Temperature

This study was made with the average monthly emissions at three temperatures, assuming linear behavior in each period. These three values (3.73 μg/L/month at 35 °C, 20.43 μg/L/month at 50 °C, and 208.5 μg/L/month at 75 °C), transformed into logarithms, were plotted against 1/*T*, giving a linear representation (log­(*k*) = 15.697–4642.9 × 1/*T*, *R* ^2^ = 0.9995, *p* < 0.05), where the slope is related with activation energy (38.6 kJ/mol). This allowed estimating that at 25 °C the emissions will be 1.19 μg/L/month and at 20 °C the emissions will be 0.64 μg/L/month. Emissions in a year at 25 °C will be 14.2, a value well above the average amounts accumulated after one year of anoxic aging, which were 1.9 μg/L.^3^ This would suggest that the fraction of MeSH emitted that reacts with the wine components is higher than that of H_2_S, as confirmed in the following section.

It is interesting to note that the slope of the log­(*k*) vs 1/*T* plot of MeSH was much lower than that of H_2_S, indicating that H_2_S emissions require higher activation energies and are more sensitive to the temperature. For practical purposes, this implies that the higher the temperature, the higher the H_2_S/MeSH ratio emitted. With our data, it could be estimated that this ratio will take the value 1 at 17.75 °C.

### 3.6 MeSH Emission and Accumulation and Methionine

The emissions of MeSH, unlike those of H_2_S, did not result in exhaustion of the MeSH precursor pool but from a very slow chemical decomposition process. The candidate precursor was methionine. However, although after 3 months of aging at 75 °C methionine decreases were significant (*p* < 0.05), they were too low (16% or 262 μg/L) to explain observed emissions. In fact, the measured decrease only accounted for 84 μg/L, less than 15% of that emitted on average in that period. Interestingly, the total MeSH emitted at 22 days at 75 °C was well correlated with the initial amount of methionine in the wine (*R* ^2^ = 0.5, *p* < 0.01), but that correlation was lost at longer times. These results suggested that free methionine was involved in MeSH emission but that it was not the main precursor. MeSH could be derived from methionine contained in proteins and could also be present in oxidized forms as the terminal part of polysulfides. It seemed unlikely that MeSH could originate from the dimethyl sulfide (DMS) precursor, S-methylmethionine, and, in fact, we saw no correlation between MeSH and DMS emissions. The other known precursors, DMDS and methylthioacetate,^16^ were found in negligible amounts to be of relevance.^1^ The nature of the MeSH precursors therefore requires further specific studies.

The ability of wine to accumulate MeSH during aging, as measured by the AR test, is significantly related to MeSH emissions. On the one hand, the BR MeSH obtained in the test was significantly and positively linearly correlated with the MeSH emitted at 35 °C in the 18 months of observation, although the slope for whites and rosés was 0.37 (*R* ^2^ = 0.7387, *p* < 0.05), while for reds it was only 0.096 (*R* ^2^ = 0.9195, *p* < 0.01). On the other hand, the free MeSH accumulated in the AR by white and rosé wines was also strongly linearly correlated with the total emitted at 35 °C, with a slope of 0.50 (*R* ^2^ = 0.9071, *p* < 0.01), while with reds, the correlation was not significant and the slope did not differ from 0. Similar relationships, not reaching statistical significance, were observed with the total MeSH emitted at 75 °C. i.e., these results indicate that the MeSH emission capacity of the wines is determinant in the accumulation of this component during anoxic aging, but that the accumulation is far more effective in whites and rosés than in reds, suggesting that part of the MeSH emitted reacts with polyphenols, which would be in agreement with recent observations about its reactivity with tannins^28^ and would confirm the observation in the previous epigraph that the amount of MeSH that accumulates is a small proportion of that emitted, less than that of H_2_S.

### 3.7 MeSH Emission and Accumulation and Dimensions of the H_2_S Pool of Precursors, [*P*]_0_

Neither the emissions nor the accumulations of MeSH and H_2_S were correlated with each other. However, both MeSH emissions and their accumulation during aging were negatively correlated with the size of the H_2_S precursor pool, [*P*]_0_. In the case of whites and rosés, both initial MeSH contents and accumulated emissions after 3 and 6 months at 35 °C were negatively correlated with [*P*]_0_ (*R* ^2^ = 0.639, *p* = 0.056). In the case of reds, emissions at 35 °C after 9 or more months were significantly and negatively correlated (*R* ^2^ = 0.671, *p* < 0.05 for 12 months). White and rosé accumulated emissions after 41 or 91 days at 75 °C were significantly and negatively correlated with [*P*]_0_ (*R* ^2^ = 0.468, *p* < 0.05; *R* ^2^ = 0.936, *p* < 0.002). The BR MeSH accumulated by the wines during RA was also significantly and negatively correlated with [*P*]_0_ (*R* ^2^ = 0.504, *p* < 0.05). Levels of free MeSH were also correlated, but statistical significance was not reached. Moreover, the BRH_2_S/BRMeSH ratio after RA was positively and significantly correlated with [*P*]_0_ (*R* ^2^ = 0.706, *p* < 0.001). Finally, the initial concentration of Met in wines was negatively and significantly correlated (*R* ^2^ = 0.496, *p* < 0.001) with *k*[*P*]_0_. With all of these results, two different hypotheses could be considered. On one hand, the competition between the MeSH emission process and the presence of H_2_S precursors could suggest that there are competitive reactions involving electron donor species in various spontaneous processes that take place during its anoxic storage.^30^ The results suggested that these electrons go primarily to the reduction of H_2_S precursors and secondarily to produce MeSH from methionine and other precursors. Indirectly, this suggests that the pool of H_2_S precursors somehow prevents the production and accumulation of MeSH during wine aging and that it plays an important role in the regulation of redox processes. On the other hand, the correlation between Met and *k*[*P*]_0_ could suggest that during the winemaking process, competitive reactions exist between the formation of Met and the formation of H_2_S precursors.

In conclusion, wines contain a pool of H_2_S precursors with defined [*P*]_0_ dimensions, capable of releasing between 1.4 and 3.0 mg/L H_2_S at high temperatures. The decomposition of these precursors follows first-order kinetics, leading to the emission and accumulation of this gas during anoxic wine aging. The kinetics are extremely temperature-dependent, consistent with previous work,^3^ and at normal temperatures are slow enough for the emissions to remain active for many years. In the first months at 35 °C, emission is correlated with GSH content.

Wine, especially red wine, also continuously emits MeSH from a pool of precursors that is only partially related to free methionine and does not appear to be depleted during the studied period. MeSH emissions are less temperature-dependent, with estimates suggesting that below 17 °C, they could be higher than those of H_2_S. MeSH emissions are responsible for its accumulation in anoxic storage, but the emitted/accumulated ratio is very low in red wines. Both the emission and accumulation of MeSH are negatively correlated with the wine’s H_2_S precursor content, [*P*]_0_, suggesting that these precursors play a key role in regulating redox processes associated with wine aging. However, the initial concentration of Met is negatively correlated with *k*[*P*]_0_, suggesting competitive synthesis reactions (Met vs H_2_S precursors) during the winemaking process.

The quantities of H_2_S and MeSH that we detected are considerably higher than those reported so far. This finding opens the door to future work aimed at building a comprehensive database of releasable H_2_S and MeSH in wines as well as identifying new precursors that could explain these amounts.

## Supplementary Material



## Notes

Transitions and collision energies (eV) used for the quantification of amino acids; chemical information on samples (metals, amino acids, and volatile sulfur compounds); characterization of samples after accelerated anoxic aging; fit plots used to interpret the emission of H_2_S and MeSH during the anoxic storage of wines at three different temperatures; and correlations between BR H_2_S accumulated after anoxic storage at 50 °C (AR) and [*P*]_0_ and free H_2_S accumulated after anoxic storage at 50 °C (AR) and H_2_S emitted at 35 °C (PDF)

## Abbreviations

H_2_S: hydrogen sulfide
MeSH: methanethiol
DMS: dimethyl sulfide
DMDS: dimethyl disulfide
[*P*]_0_: total H_2_S precursors
BR: brine-releasable
AR: accelerated reductive aging
Cys: cysteine
Met: methionine
GSH: glutathione
HPLC-MS: high-performance liquid chromatography–mass spectrometry
HS-GC-SCD: headspace-gas chromatography-sulfur chemiluminescence detector
