# Supplementary material for: Kinetic Studies on the Ability of Wines to Produce Hydrogen Sulfide (H2S) and Methanethiol (MeSH)
Source: J Agric Food Chem. 2025 Jul 28;73(31):19684–92. doi: 10.1021/acs.jafc.5c04010 (PMC12333344; doi:10.1021/acs.jafc.5c04010)
Supplement: Supplementary file 1 [file jf5c04010_si_001.pdf]

Kinetic studies on the ability of wines to produce hydrogen sulfide (H_2S) and methanethiol (MeSH)

Susana Ainsa-Zazurca, Ignacio Ontañón and Vicente Ferreira*

Laboratory for Flavor Analysis and Enology (LAAE), Department of Analytical Chemistry, Faculty of Sciences, AgriFood Institute of Aragon-IA2 (University of Zaragoza-CITA), Zaragoza, 50009, Spain.

*+34-976762067; vferre@unizar.es

Table S1. Transitions and collision energies (eV) used for the quantification of amino acids.

Amino acid	Precursor ion (m/z)	Collision energy (eV)	Product ion (m/z)
Cys	122	10	105
			76
Met	150	10	104
		8	133
GSH	308	10	179
			104

Table S2. Chemical information of samples (metals and amino acids).

	Zn 68 (µg/L)	Zn 67 (µg/L)	Cu 63 (µg/L)	Cu 65 (µg/L)	Mn 55 (µg/L)	Fe 56 (µg/L)	Fe 57 (µg/L)	Cys (µg/L)	Met (µg/L)	GSH (µg/L)
**VT1**	528 ± 138	542 ± 148	47.6 ± 0.4	47.7 ± 0.1	3,300 ± 61	1,120 ± 1	1,120 ± 8	<LOD	816 ± 37	43.6 ± 6.1
**VT2**	295 ± 314	294 ± 314	116 ± 0	116 ± 0	648 ± 1	2,020 ± 10	2,040 ± 14	<LOD	1372 ± 21	26.4 ± 7.1
**VT3**	863 ± 248	863 ± 251	64.1 ± 0.2	64.4 ± 0.3	729 ± 9	1,730 ± 9	1,730 ± 4	<LOD	1035 ± 14	14.3 ± 5.6
**VT4**	430 ± 52	432 ± 49	39.7 ± 0.4	39.5 ± 0.2	868 ± 0	1,500 ± 8	1,500 ± 18	<LOD	1359 ± 34	55.7 ± 13.6
**VT5**	511 ± 115	512 ± 114	25.5 ± 0	25.5 ± 0.2	793 ± 7	1,560 ± 5	1,550 ± 8	<LOD	2333 ± 26	37.4 ± 9.2
**VT6**	672.87 ± 56	665 ± 57	29.7 ± 0.3	29.6 ± 0.1	1,680 ± 3	846 ± 4	847 ± 13	<LOD	1947 ± 97	27.04 ± 3.58
**VB7**	575 ± 221	580 ± 217	6.71 ± 0.04	6.62 ± 0.11	1,040 ± 3	1,100 ± 1	1,110 ± 7	<LOD	1860 ± 107	50.3 ± 30.8
**VB8**	998 ± 221	992 ± 223	53.5 ± 0.5	53.2 ± 0.4	923 ± 3	4,470 ± 22	4,490 ± 28	<LOD	1881 ± 108	21.8 ± 1.6
**VB9**	611 ± 122	606 ± 126	33.9 ± 0.3	34.3 ± 0.2	1,310 ± 4	981 ± 8	982 ± 9	<LOD	3047 ± 161	28.8 ± 5.0
**VR10**	823 ± 458	816 ± 456	4.89 ± 0.07	4.69 ± 0.15	722 ± 1	664 ± 1	667 ± 6	<LOD	1621 ± 54	21.7 ± 7.1
**VR11**	30.4 ± 0.8	29.4 ± 1.4	266 ± 2	269 ± 2	990 ± 2	1,270 ± 8	1,270 ± 13	<LOD	1394 ± 49	19.5 ± 5.3
**VB12**	33.4 ± 42	33.0 ± 43	9.15 ± 0.44	8.95 ± 0.34	852 ± 3	1,850 ± 2	1,850 ± 11	<LOD	1785 ± 76	27.2 ± 6.7

LOD Cys: 60 µg/L.

Table S3. Chemical information of samples (volatile sulfur compounds).

	**Free H_2S (µg/L)**	**Free SO_2 (mg/L)**	**Free MeSH (µg/L)**	**Free EtSH (µg/L)**	**Free DMS (µg/L)**	**Free DMDS (µg/L)**	**BR H_2S (µg/L)**	**BR MeSH (µg/L)**	**BR EtSH (µg/L)**	**BR DMS (µg/L)**	**BR DMDS (µg/L)**
**VT1**	0.110 ± 0.004	6.94 ± 0.51	<LOD	<LOD	17.7 ± 0.2	<LOD	30.5 ± 7.8	3.062 ± 1.156	<LOD	19.2 ± 0.2	<LOD
**VT2**	2.56 ± 0.8	2.85 ± 0.19	0.966 ± 1.317	<LOD	29.01 ± 1.89	<LOD	14.3 ± 2.2	5.59 ± 0.68	<LOD	29.1 ± 1.6	<LOD
**VT3**	0.290 ± 0.002	3.77 ± 1.69	<LOD	<LOD	29.7 ± 0.5	<LOD	32.8 ± 1	2.92 ± 0.44	<LOD	29.5 ± 0.2	<LOD
**VT4**	0.832 ± 0.122	3.84 ± 1.58	0.577 ± 0.77	<LOD	101 ± 0	<LOD	10.9 ± 4.9	3.91 ± 0.64	<LOD	126 ± 1	<LOD
**VT5**	1.73 ± 0.5	9.75 ± 1.07	1.54 ± 0.11	<LOD	52.8 ± 1.1	0.248 ± 0.21	19.1 ± 1.2	5.30 ± 0.58	<LOD	46.1 ± 0	<LOD
**VT6**	6.83 ± 0.13	1.69 ± 0.04	2.61 ± 0	<LOD	39.7 ± 0.2	0.218 ± 0.17	13.3 ± 1.6	7.82 ± 0.09	<LOD	28.7 ± 0.1	<LOD
**VB7**	3.45 ± 0.05	31.1 ± 2.2	0.939 ± 1.28	<LOD	22.4 ± 0.5	1.03 ± 0.07	22.7 ± 8	3.95 ± 0.04	<LOD	23.9 ± 0.1	<LOD
**VB8**	7.61 ± 5.85	36.03 ± 1.68	2.28 ± 0.33	<LOD	15.0 ± 0.3	0.309 ± 0.3	9.57 ± 3.37	5.23 ± 2.01	<LOD	17.02 ± 0.18	<LOD
**VB9**	0.535 ± 0.393	18.2 ± 0	5.44 ± 0.48	<LOD	39.8 ± 0.6	<LOD	24.9 ± 6.9	8.40 ± 0.43	<LOD	43.7 ± 0.3	<LOD
**VR10**	0.792 ± 0.423	17.6 ± 0.3	1.36 ± 0.08	<LOD	104 ± 2	<LOD	31.1 ± 0.8	0.839 ± 0.278	<LOD	112 ± 2	<LOD
**VR11**	8.44 ± 4.96	15.8 ± 1.7	3.30 ± 1.16	<LOD	39.0 ± 1.7	<LOD	29.1 ± 10.6	5.37 ± 0.49	<LOD	41.4 ± 1.3	<LOD
**VB12**	0.0152 ± 0.0152	20.5 ± 0	<LOD	<LOD	35.5 ± 0.1	<LOD	92.1 ± 7.7	<LOD	<LOD	39.8 ± 0.7	<LOD

LOD Free MeSH: 0.035 µg/L; LOD Free EtSH: 0.37 µg/L; LOD Free DMDS: 0.1 µg/L; LOD BR MeSH: 0.35 µg/L; LOD BR EtSH: 0.4 µg/L; LOD BR DMDS: 0.7 µg/L.

Table S4. Characterization of samples after accelerated anoxic aging (redox potential, content of volatile sulfur compounds and amino acids).

	Redox potential (mV)	Free H_2S (µg/L)	Free SO_2 (mg/L)	Free MeSH (µg/L)	Free EtSH (µg/L)	Free DMS (µg/L)	Free DMDS (µg/L)	BR H_2S (µg/L)	BR MeSH (µg/L)	BR EtSH and DMDS (µg/L)	BR DMS (µg/L)	Cys (µg/L)	Met (µg/L)	GSH (µg/L)
**VT1**	-23 ± 7	30.3 ± 0.4	1.99 ± 0.14	4.82 ± 0.2	<LOD	48.8 ± 0.7	<LOD	68.9 ± 7	8.76 ± 0.29	<LOD	47.6 ± 1.8	<LOD	863 ± 26	<LOD
**VT2**	-59 ± 1	25.3 ± 0.4	0.586 ± 0.059	5.87 ± 0.19	<LOD	54.5 ± 0.7	<LOD	44.5 ± 4.1	9.80 ± 0.91	<LOD	53.2 ± 0.7	<LOD	1128 ± 70	4.60 ± 4.04
**VT3**	-29 ± 26	11.3 ± 0.7	1.43 ± 0.13	2.33 ± 0.54	<LOD	54.9 ± 0.7	<LOD	85.4 ± 24.6	9.25 ± 5.17	<LOD	53.4 ± 9.2	<LOD	704 ± 49	0.715 ± 1.238
**VT4**	-80 ± 8	20.5 ± 4.2	1.91 ± 0.29	4.22 ± 0.14	<LOD	390 ± 0	<LOD	63.2 ± 19.2	8.16 ± 2.67	<LOD	425 ± 48	<LOD	1101 ± 45	<LOD
**VT5**	-120 ± 44	19.7 ± 0.7	2.52 ± 0.21	3.87 ± 0.22	<LOD	100 ± 1	<LOD	42.9 ± 1.6	11.3 ± 0.3	<LOD	99.8 ± 0.9	<LOD	1844 ± 284	<LOD
**VT6**	-190 ± 2	21.1 ± 0.5	0.623 ± 0.167	6.37 ± 0.49	<LOD	71.02 ± 0.44	<LOD	40.9 ± 3.3	14.3 ± 0.7	<LOD	68.1 ± 5.4	<LOD	1008 ± 849	6.17 ± 10.68
**VB7**	-160 ± 25	20.3 ± 0.3	26.3 ± 0.9	3.12 ± 0.68	<LOD	50.0 ± 0.4	0.602 ± 0.4	56.7 ± 6.3	11.9 ± 0.6	<LOD	50.0 ± 1.3	<LOD	1865 ± 86	10.7 ± 10.9
**VB8**	-190 ± 11	10.8 ± 0.5	29.6 ± 0.4	3.031 ± 0.345	<LOD	39.5 ± 0.6	0.649 ± 0.39	35.2 ± 5.8	12.2 ± 0.4	<LOD	40.5 ± 1.6	<LOD	1533 ± 39	4.14 ± 3.63
**VB9**	-76 ± 6	8.012 ± 0.331	6.88 ± 2.18	16.3 ± 0.3	<LOD	85.4 ± 1.4	0.561 ± 0.349	40.7 ± 0.7	20.4 ± 0.4	<LOD	80.3 ± 0.8	<LOD	3365 ± 579	<LOD
**VR10**	-84 ± 3	23.6 ± 0.5	8.73 ± 1.63	7.067 ± 0.126	2.89 ± 5.71	320 ± 6	<LOD	47.7 ± 3.6	9.23 ± 0.82	<LOD	288 ± 11	<LOD	917 ± 49	9.35 ± 1.37
**VR11**	-84 ± 4	24.8 ± 1.3	13.8 ± 0.4	10.2 ± 0.8	2.65 ± 3	120 ± 5	0.871 ± 0.296	41.0 ± 6.1	14.8 ± 0.5	<LOD	115 ± 3	<LOD	1363 ± 39	7.67 ± 1.17
**VB12**	-92 ± 18	9.15 ± 5.62	15.3 ± 0.9	2.66 ± 1.93	<LOD	83.01 ± 0.39	0.681 ± 0.223	149 ± 4	8.028 ± 0.352	<LOD	79.7 ± 1.6	<LOD	1614 ± 108	10.4 ± 0.5

LOD Free EtSH: 0.37 µg/L; LOD Free DMDS: 0.1 µg/L; LOD BR EtSH: 0.4 µg/L; LOD BR DMDS: 0.7 µg/L; LOD Cys: 60 µg/L; LOD GSH: 30 µg/L.

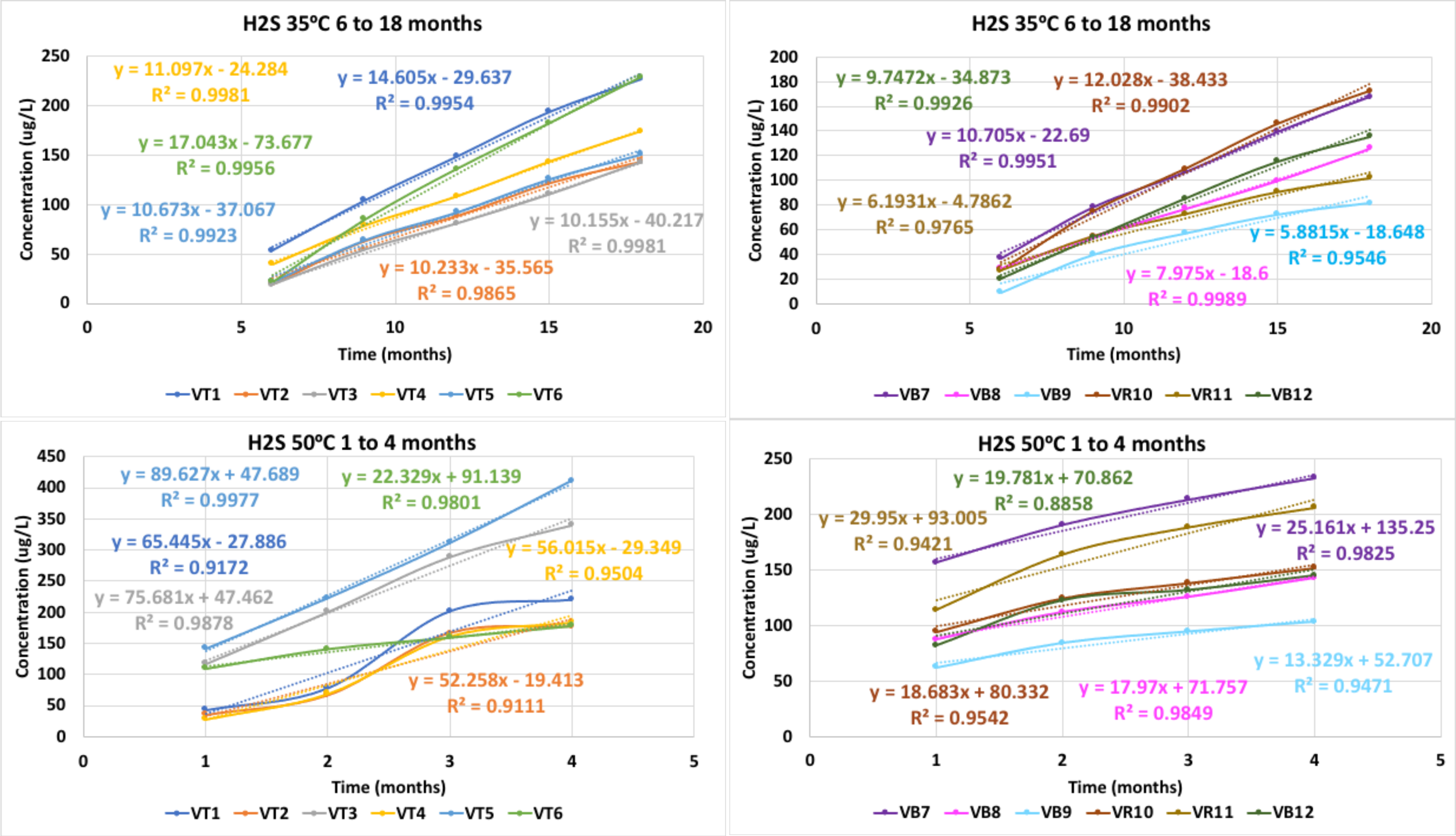
H2S 35ºC 6 to 18 months
Concentration (ug/L)
Time (months)
y = 11.097x - 24.284
R² = 0.9981
y = 14.605x - 29.637
R² = 0.9954
y = 17.043x - 73.677
R² = 0.9956
y = 10.673x - 37.067
R² = 0.9923
y = 10.155x - 40.217
R² = 0.9981
y = 10.233x - 35.565
R² = 0.9865
VT1 VT2 VT3 VT4 VT5 VT6
H2S 35ºC 6 to 18 months
Concentration (ug/L)
Time (months)
y = 9.7472x - 34.873
R² = 0.9926
y = 12.028x - 38.433
R² = 0.9902
y = 10.705x - 22.69
R² = 0.9951
y = 6.1931x - 4.7862
R² = 0.9765
y = 5.8815x - 18.648
R² = 0.9546
y = 7.975x - 18.6
R² = 0.9989
VB7 VB8 VB9 VR10 VR11 VB12
H2S 50ºC 1 to 4 months
Concentration (ug/L)
Time (months)
y = 89.627x + 47.689
R² = 0.9977
y = 22.329x + 91.139
R² = 0.9801
y = 65.445x - 27.886
R² = 0.9172
y = 56.015x - 29.349
R² = 0.9504
y = 75.681x + 47.462
R² = 0.9878
y = 52.258x - 19.413
R² = 0.9111
VT1 VT2 VT3 VT4 VT5 VT6
H2S 50ºC 1 to 4 months
Concentration (ug/L)
Time (months)
y = 19.781x + 70.862
R² = 0.8858
y = 29.95x + 93.005
R² = 0.9421
y = 25.161x + 135.25
R² = 0.9825
y = 13.329x + 52.707
R² = 0.9471
y = 18.683x + 80.332
R² = 0.9542
y = 17.97x + 71.757
R² = 0.9849
VB7 VB8 VB9 VR10 VR11 VB12

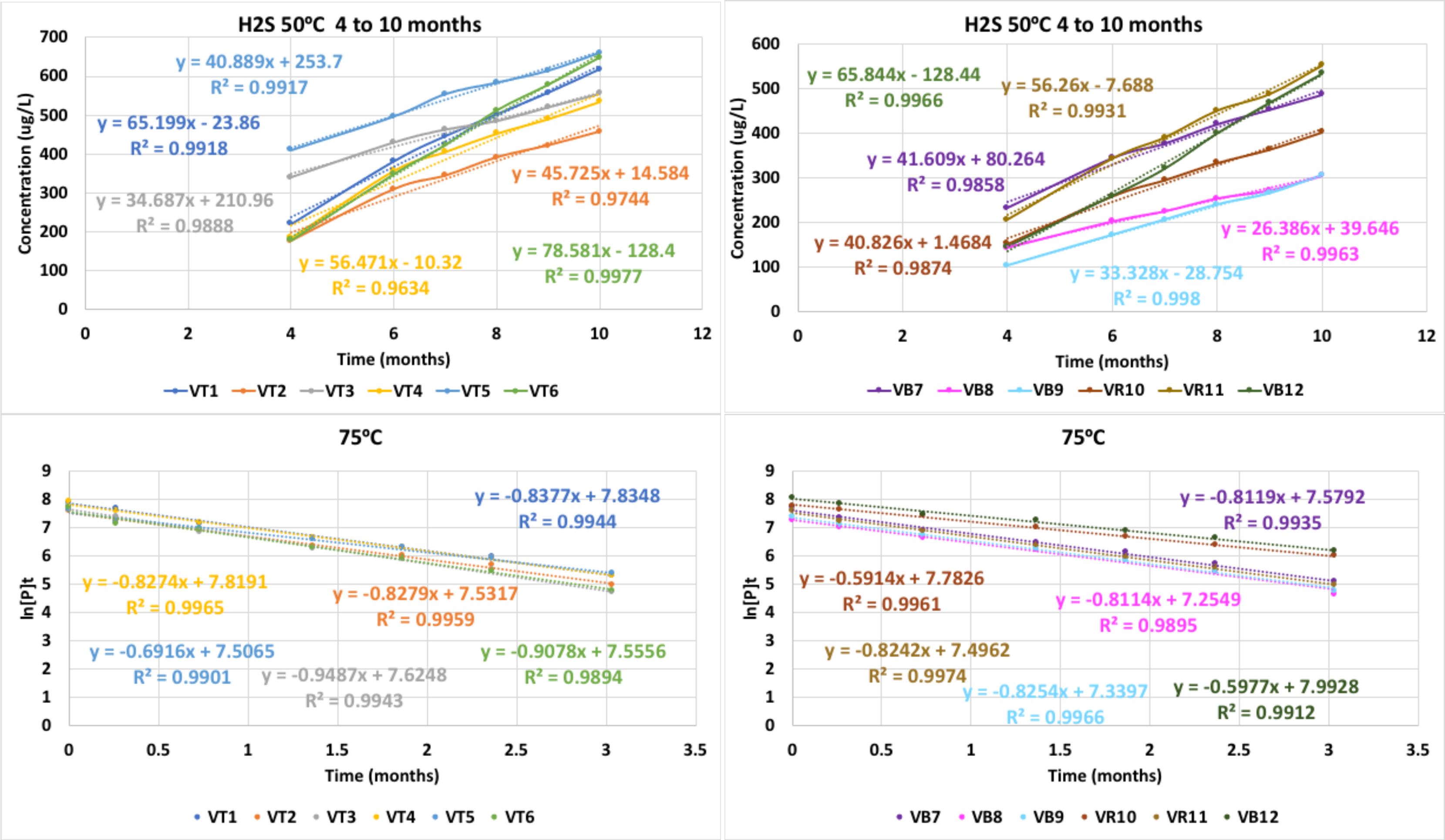

Figure S1. Fit plots used to interpret the emission of H_2S during the anoxic storage of wines at three different temperatures (Complementary information to Table 2).

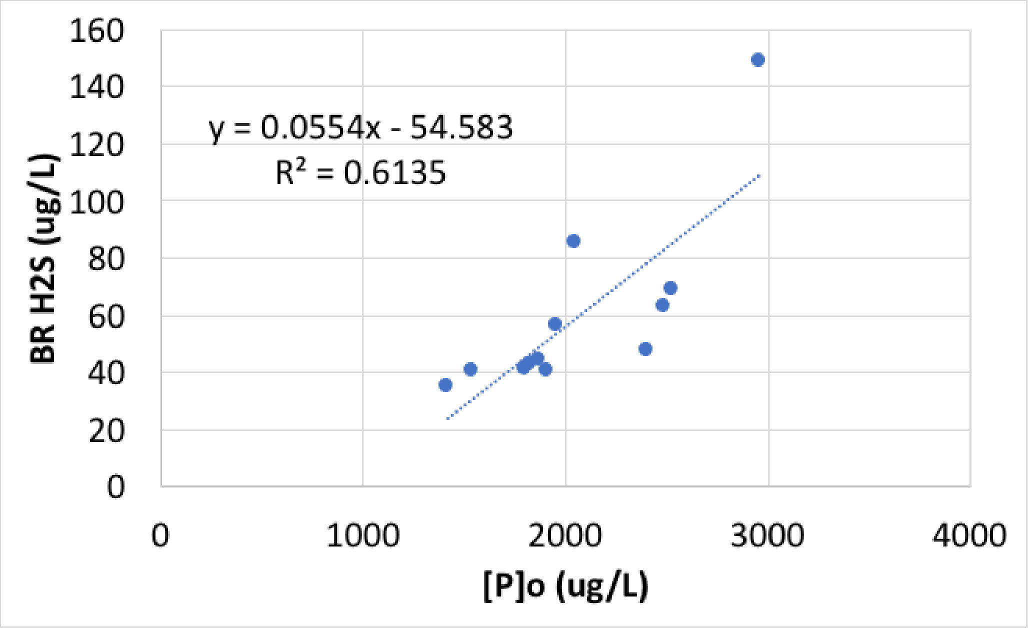

Figure S2. Correlation between BR H_2S accumulated after anoxic storage at 50°C (AR) and $[P]_0$.

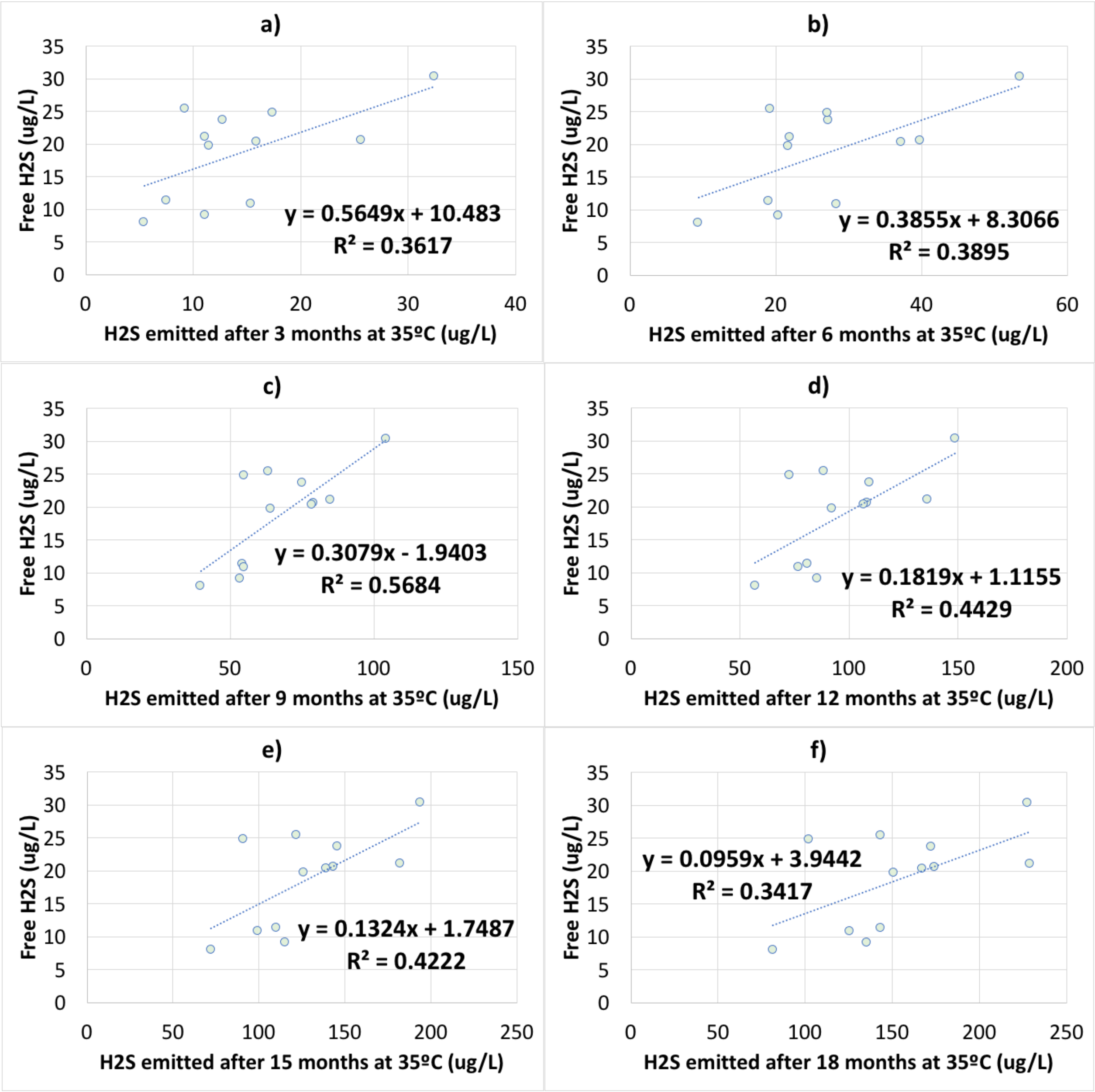

Figure S3. Correlations between free H_2S accumulated after anoxic storage at 50°C (AR) and H_2S emitted at 3, 6, 9, 12, 15 or 18 months, a to f respectively, at 35°C.

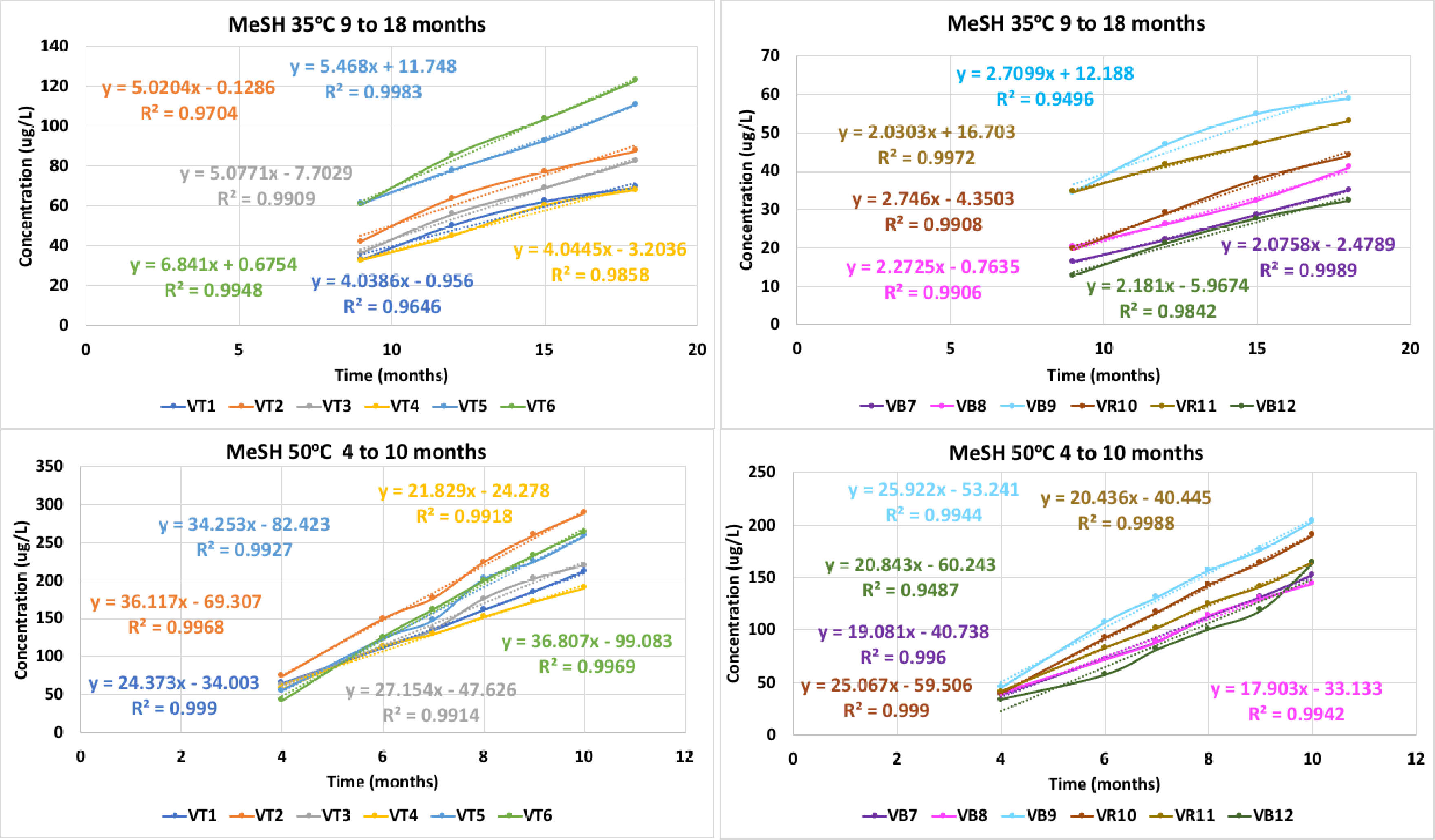

Figure S4. Fit plots used to interpret the emission of MeSH during the anoxic storage of wines at three different temperatures (Complementary information to Table 3).
